# Chlorin e6-Coated Superparamagnetic Iron Oxide Nanoparticle (SPION) Nanoclusters as a Theranostic Agent for Dual-Mode Imaging and Photodynamic Therapy

**DOI:** 10.1038/s41598-019-39036-1

**Published:** 2019-02-22

**Authors:** Ahmad Amirshaghaghi, Lesan Yan, Joann Miller, Yonathan Daniel, Joel M. Stein, Theresa M. Busch, Zhiliang Cheng, Andrew Tsourkas

**Affiliations:** 1 Department of Bioengineering, University of Pennsylvania, Philadelphia, PA 19104 USA; 2 Department of Radiation Oncology, Perelman School of Medicine, University of Pennsylvania, Philadelphia, PA 19104 USA; 3 Department of Biology, College of Computer, Mathematical, & Natural Sciences, University of Maryland, College Park, Maryland 20742 USA; 4 Department of Radiology, University of Pennsylvania, Philadelphia, PA 19104 USA

## Abstract

Photodynamic therapy (PDT) is an approved modality for the treatment of various types of maligancies and diseased states. However, most of the available photosensitizers (PS) are highly hydrophobic, which limits their solubility and dispersion in biological fluids and can lead to self-quenching and sub-optimal therapeutic efficacy. In this study, chlorin e6 (Ce6)-coated superparamagnetic iron oxide nanoparticle (SPION) nanoclusters (Ce6-SCs) were prepared via an oil-in-water emulsion. The physical-chemical properties of the Ce6-SCs were systematically evaluated. Dual-mode imaging and PDT was subsequently performed in tumor-bearing mice. Chlorin e6 is capable of solubilizing hydrophobic SPION into stable, water-soluble nanoclusters without the use of any additional amphiphiles or carriers. The method is reproducible and the Ce6-SCs are highly stable under physiological conditions. The Ce6-SCs have an average diameter of 92 nm and low polydispersity (average PDI < 0.2). Encapsulation efficiency of both Ce6 and SPION is ≈100%, and the total Ce6 payload can be as high as 56% of the total weight (Ce6 + Fe). The Ce6-SCs localize within tumors via enhanced permeability and retention and are detectable by magnetic resonance (MR) and optical imaging. With PDT, Ce6-SCs demonstrate high singlet oxygen generation and produce a significant delay in tumor growth in mice.

## Introduction

Photodynamic therapy (PDT) is a minimally-invasive procedure for the treatment of cancers. PDT uses light irradiation in combination with chemical photosensitizers (PS) to eradicate target tumor tissues. In the absence of light, PS are nontoxic to cells, but when illuminated with specific activating wavelengths, the photosensitizers generate cytotoxic reactive oxygen species (ROS) that destroy cells^1–5^. Compared with ionizing radiation therapy or chemotherapy, PDT can be safer for the surrounding normal tissues or organs because the generation of ROS is a light-triggered process^6^, thus limiting the area of exposure, and photosensitizers can preferentially accumulate in tumor cells^7^, further improving the specificity of therapy. PDT has several advantages over more conventional cancer therapies, including cost-effectiveness, highly localized and specific tumor treatments, outpatient therapy, and higher cure rates for some tumors^8,9^. In several studies, photosensitizers were combined with magnetic fluids (or magnetic resonance, MR, contrast agents) for combined MR imaging and photodynamic therapy^10–12^.

The efficacy of PDT depends on the photosensitizing agent, its concentration, as well as the cell type^13^. PS dosage is severely limited by the poor water solubility of most PS agents. Moreover, many of the clinically-used PS molecules are excited by visible light with limited tissue penetration^14–16^ and show limited selecvity for tumor tissue, making treatment damage to normal tissue a key concern^17,18^. The use of long-wavelength laser irradiation (650–900 nm) significantly improves the depth penetration for *in vivo* PDT^19^, while the incorporation of PS agents into nanoparticles offers an opportunity to improve PS solubility and reduce accumulation and damage in normal tissues^1,20–22^.

Chlorin e6 (Ce6) is a second generation and clinically-used photosensitizer that is characterized by high sensitizing efficacy and rapid elimination from the body. Ce6 can be excited with a 660–670 nm laser that can penetrate deeper into human tissue than the 630-nm laser used for conventional or first generation photosensitizers such as Photofrin^23–26^. For example, 665 nm light penetrates 22% deeper than 633 nm light in the human prostate gland^27^. When irradiated, Ce6 has a high singlet oxygen (^1^O_2_) quantum yield and shows low dark toxicity, which makes Ce6 a favorable PS for PDT. Promising clinical benefits have been obtained with Ce6-mediated PDT (Ce6-PDT) for the treatment of lung, bladder, skin and head and neck cancers^1,4,14^. Moreover, Ce6 exhibits improved therapeutic efficacy and reduced side effects compared to conventional photosensitizers that stem from hematoporphyrin derivatives^28^. However, the clinical use of Ce6 has primarily been limited by its poor water solubility^2^. Furthermore, sharp Soret and Q bands are observed for Ce6 in protic solvents except for water^14,29^. To improve the poor water solubility of Ce6 for PDT, various kinds of nano-sized drug carriers such as nano-graphene^20^ and gold vesicles^21^, or PS-conjugates with polyvinylpyrrolidone (PVP)^24,30,31^, human serum albumin^2^, polymeric micelles^32^, silica^22^, peptides^33^, glucamine (BLC 1010)^34^, and Ce6-conjugates with superparamagnetic iron oxide nanoparticles (SPIONs) by multistep chemical reactions^35–37^ have been developed. Unfortunately, Ce6’s characteristic PDT properties are often suppressed when incorporated into nanocarriers due to quenching^38–41^. Moreover, scaling up the synthesis and achieving a reproducible manufacturing process can be a major challenge. Therefore, there is still a need to develop new Ce6 formulations that are stable, scalable, reproducible and capable of delivering Ce6 to tumors in an efficient manner, without compromising its PDT properties.

Recently, we discovered that amphiphilic small molecule dyes can be used to solubilize nanoclusters of superparamagnetic iron oxide nanoparticles (SPIONs)^42–44^. In this study, we demonstrate that Ce6 can also be used to solubilize nanoclusters of SPIONs without using any extra carrier or complicated chemical reaction. Ce6 is structurally similar to PpIX^42^, but possesses a higher peak extinction coefficient (55,000 at 667 nm vs. 5,186 at 631 nm), has a 46 nm red-shifted peak absorbance, and higher singlet oxygen quantum yield (0.75 vs. 0.54). The physical-chemical properties of the Ce6-SPION clusters (Ce6-SCs) were systematically evaluated. The Ce6-SCs were further tested for their ability to serve as magnetic resonance and fluorescence contrast agent as well as a PS for PDT in a murine tumor model.

## Results and Discussion

### Synthesis and physical-chemical characterization of Ce6-SCs

**Figure 1:**
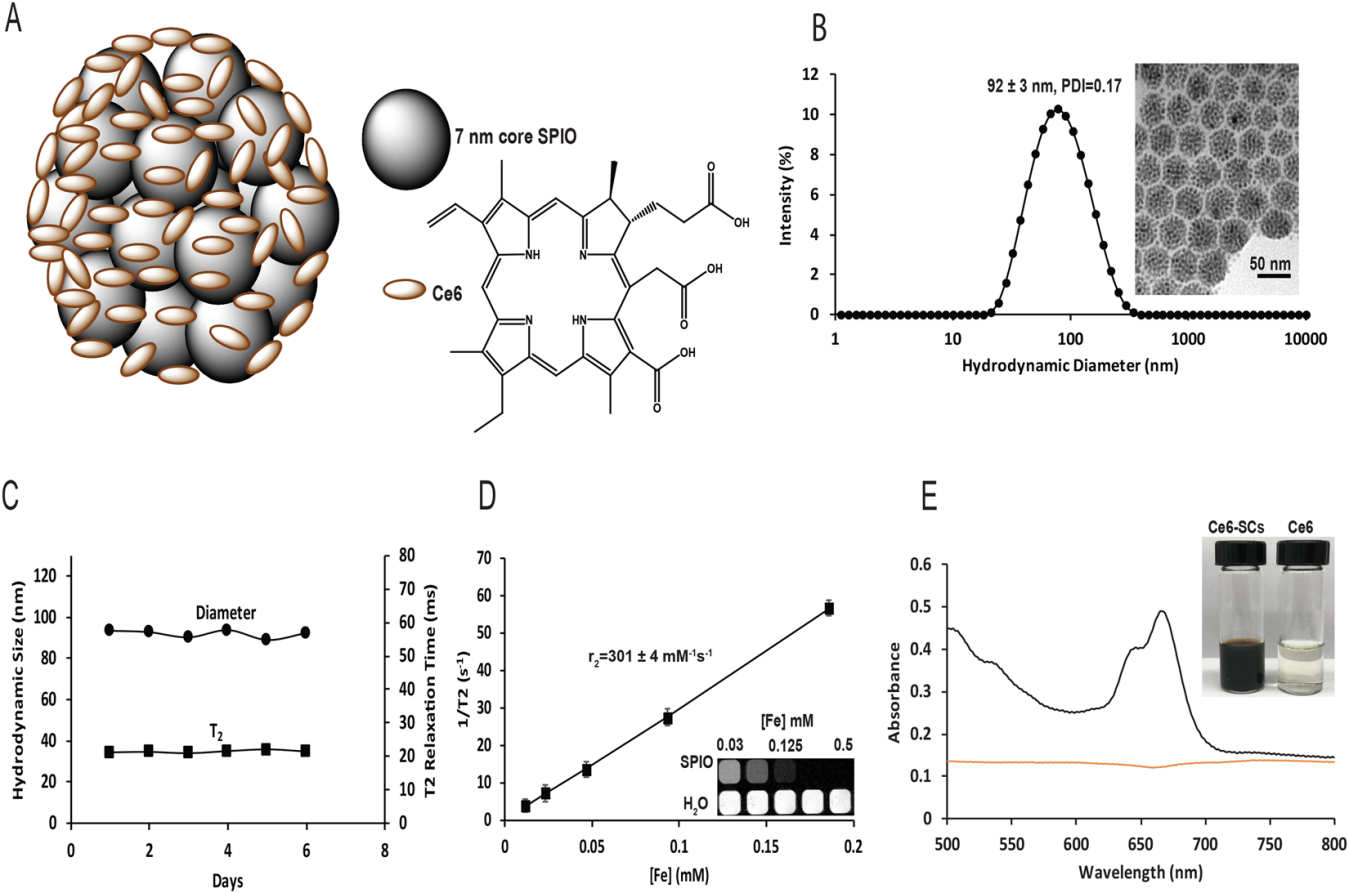
(**A**) Illustration of Ce6-coated SPION nanoclusters (Ce6-SCs). Iron oxide nanoparticles are stabilized in aqueous media by the self-assembly of amphiphilic PS Chlorin e6 on the surface using a microemulsion. (**B**) Dynamic light scattering (DLS) of Ce6-SCs in water. Inset: TEM image of Ce6-SCs shows tightly packed nanoclusters (scale bar: 50 nm). (**C**) Particle size and T_2_ relaxation time was monitored for 6 days in water at 25 °C. (**D**) Magnetic resonance (MR) relaxometry measurements of Ce6-SCs. Inset: T_2_-weighted image of Ce6-SCs at various concentrations in a microplate. (**E**) Absorbance spectra of Ce6-SCs (black) and free Ce6 (brown) in water. The inset images are vials containing solutions of Ce6-SCs and free Ce6 in water.

Ce6-SCs (Fig. 1A) were prepared by simply dissolving two clinically-used functional materials, Ce6 and SPIONs (diameter = 7.6 ± 1.0 nm; Fig. S1, Supplementary information) at a ratio of 2:1, into the oil phase of an oil-in-water emulsions. No additional amphiphiles or carrier materials were required. The amphiphilic Ce6 molecules solubilized the hydrophobic SPIONs, creating stable nanoclusters with an average hydrodynamic diameter of 96.38 ± 4.6 nm and an average polydispersity index (PDI) of <0.2 (Fig. 1B). Transmission electron microscopy (TEM) images confirmed the formation of tightly packed SPIONs nanoclusters with a narrow size distribution (Fig. 1B, inset). The preparation of Ce6-SCs was highly reproducible (Table S1, Supplementary information) and easily scalable.

The effect of varying the amount of Ce6, with a fixed amount of SPIONs, on the physical-chemical properties of the Ce6-SCs was evaluated. All measurements were made after free Ce6 was removed by magnetic purification. It was found that the encapsulation efficiency is >96% for SPION and >90% for Ce6, when the Ce6:Fe ratio (w/w) is in the range of 1:3 to 1:2.5 (Table S2, Supplementary information), which is noticeably higher than most other methods that have been used to solubilize Ce6^2,6,21,35,37^. The Ce6 payload was approximately 25% of the total weight (Ce6 + Fe) at these same ratios. Increasing the Ce6:Fe ratio to 2:1 led to a reduction in encapsulation efficiency to 65%, but the payload increased to 56% of the total weight. In general, the hydrodynamic diameter increased as the Ce6:Fe ratio decreased. The PDI was <0.2 for all conditions tested. The r_2_ relaxivity of the Ce6-SCs also increased, from 247 mM^−1^ s^−1^ to 410 mM^−1^ s^−1^, as the Ce6:Fe ratio decreased from 2:1 to 1:3. This is likely the result of an increase in the number of SPIONs per cluster. Ce6-SCs formed using a Ce6:Fe ratio of 1:1 were used for all subsequent studies, since they provided balanced values for encapsulation efficiency, payload, and relaxivity.

### Stability of Ce6-SCs

Ce6-SCs demonstrated size stability in water without aggregation or precipitation as indicated by no significant changes in the T_2_ relaxation time (Fig. 1C) or hydrodynamic diameter over the course of at least 6 days. Relaxometry measurements indicated an average r_2_ value of 301 ± 4 mM^−1^ s^−1^ and MR images of a phantom confirmed strong T_2_ contrast relative to water (Fig. 1D). Analysis of the absorbance spectrum of Ce6-SCs reveals a distinct peak at ~670 nm, suggestive of excellent solubility. In comparison, there was no absorbance peak for free Ce6 (Fig. 1E, inset and Fig. S2B,D, Supplementary information), owing to its poor solubility and susceptibility to aggregation. Not surprisingly, the loading of the high payload of Ce6 on nanoclusters does result in decreased fluorescent intensities, due to both self-quenching and iron-mediated quenching (Fig. S2A, Supplementary information). The degree of fluorescence quenching for Ce6-SCs is ~90% in water and ~48% in PBS, compared to nanoclusters that have been completely dissolved in DMSO (Fig. S2C, Supplementary information). Interestingly, the fluorescence of Ce6-SCs is only quenched ~15% compared with free Ce6, when both are in PBS (Fig. S2C,E, Supplementary information).

**Figure 2:**
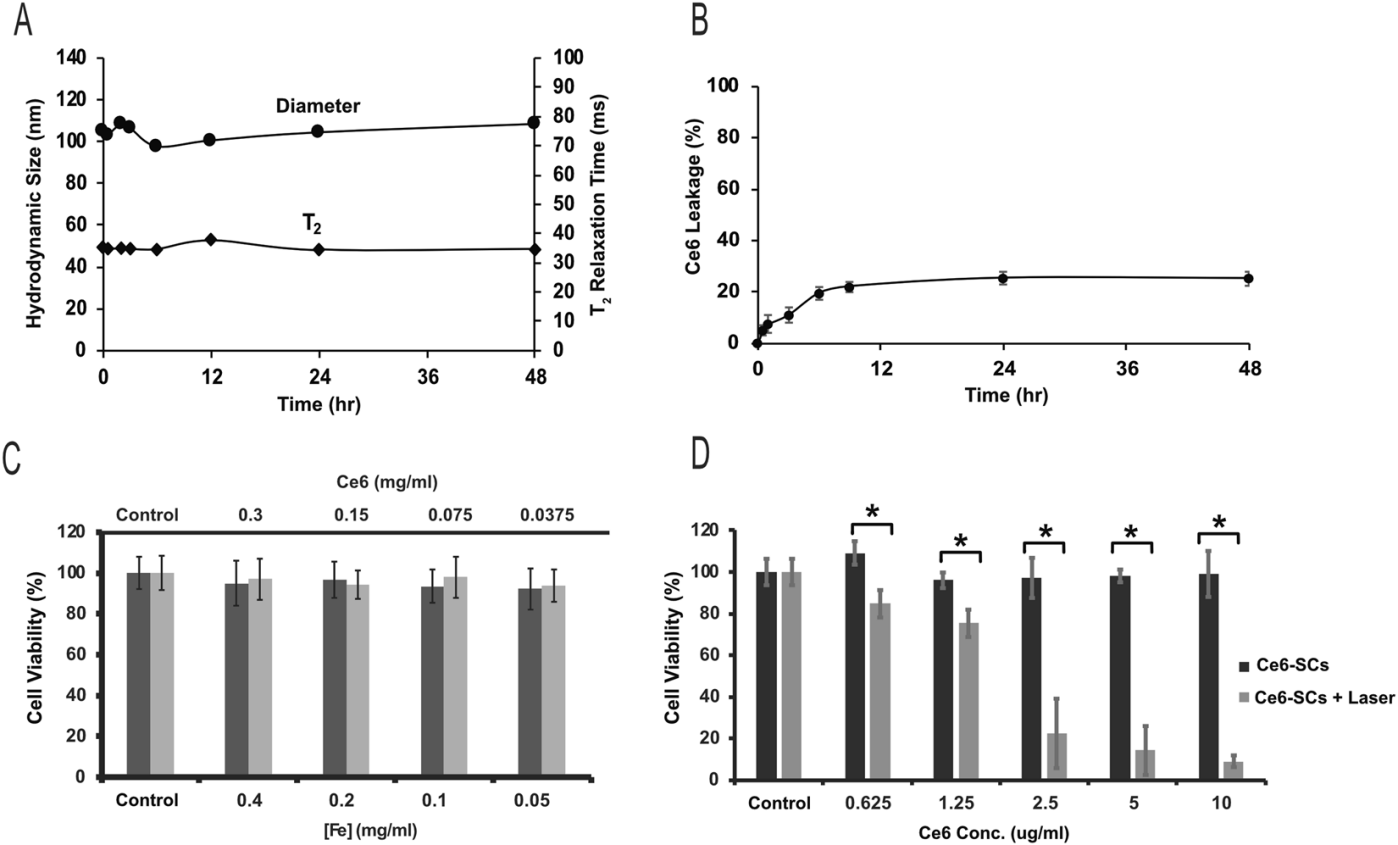
(**A**) Ce6-SCs were incubated in serum, at 37 °C. Hydrodynamic size and T_2_ relaxation time were monitored as a function of time. (**B**) Ce6-SCs in serum (37 °C) were purified by magnetic separation at various time points to quantify the rate of Ce6 release/dissociation. (**C**) Viability of HUVEC cells (black bar) and 4T1 cells (gray bar) after incubation with increasing concentrations of Ce6-SCs for 24 h. (**D**) Viability of 4T1 cells treated with different concentrations of Ce6-SCs, with and without laser irradiation (665 nm, 5 J/cm^2^) (*P < 0.0001, unpaired *t* test).

The stability of Ce6-SCs was tested by incubating Ce6-SCs in FBS for 48 h at 37 °C under constant shaking. The hydrodynamic diameter of Ce6-SCs increased by 10–15 nm immediately upon addition to serum, due to protein absorption, but then remained constant over the remaining 48 h. No aggregation or precipitate was observed and there was no remarkable change in the T_2_ relaxation time (Fig. 2A). The release of Ce6 from Ce6-SCs was evaluated by magnetic separation. As shown in Fig. 2B, the percentage of Ce6 leaking from the nanoparticles was only about 22% within 9 h after addition of serum, and then no further release was detectable for up to 48 h. These results confirm that the Ce6 forms a highly stable interaction with the SPION surface.

### Singlet oxygen production with Ce6-SCs

To evaluate the singlet oxygen (^1^O_2_) generation efficiency of Ce6-SCs, compared to that of free Ce6, we used ABDA (9,10-anthracenediylbis(methylene) dimalonic acid) - an establshed photochemical probe for singlet oxygen and other reactive oxygen species (ROS). Optical absorption spectra of ABDA changed as a function of time in response to irradiation of ABDA-containing samples of Ce6-SCs as well as of neat Ce6 at 665 nm (5 mW/cm^2^) (Fig. S3, Supplementary information). Specifically, the absorbance of ABDA decreased rapidly for both Ce6-SCs and free Ce6. Incorporation of Ce6 into the nanoclusters resulted in little to no change in the response of ABDA. The response was observed whether Ce6-SCs were in water or DMSO. These findings suggest that the ability to generate singlet oxygen by Ce6 is not significantly altered when the chromophores are incorporated into Ce6-SCs, in spite of the Ce6 molecules being densely packed on the surface of SPIONs. We attempted to compare the singlet oxygen production of Ce6-SCs with Ce6 that had been encapsulated in micelles, to see if the lack of quenching was unique to Ce6-SCs, due to the likely alignment of the amphiphilic Ce6 with its hydrophobic domains facing the SPIONs and its hydrophilic domains facing the surrounding water. In micelles, Ce6 is likely to be randomly oriented. Unfortunately, encapsulation of Ce6 in micelles was not successful due to the amphiphilic nature of Ce6.

To explore how the singlet oxygen production by Ce6 and Ce6-SCs compares to that by Protoporphyrin IX (PpIX), a common PS with chemical structure similar to that of Ce6, and by PpIX-coated SPION nanoclusters, we monitored the absorption spectrum of ABDA when irradiating these samples at 632 nm (5 mW/cm^2^) - the peak absorption of PpIX (Fig. S4, Supplementary information). Like in the case of Ce6, significant singlet oxygen production was observed by both free PpIX and PpIX-SCs, with PpIX-SCs only exhibiting a slightly lower level of singlet oxygen production. However, when PpIX was encapsulated within micelles, no singlet oxygen production was observed upon irradiation.

In our system we observe that the fluorescence of Ce6 is strongly quenched when it is packed on the surface of the nanoclusters. This behavior is consistent with the ability to generate singlet oxygen, provided the reduction in fluorescence is caused by an enhancement of intersystem crossing and subsequent formation of the triplet state. In this regard, the proximity of the chromophores to the iron ions on the SPION surfaces may have enhanced spin-orbit coupling due to the external heavy atom effect^45,46^, thereby increasing triplet formation. Fluorescence quenching also could be a result of exciton coupling between two nearby Ce6 molecules. It is known that intersystem crossing to the triplet state can be enhanced in exciton coupled systems with parallel of oblique orientation of transition dipole moments due to the forbiddance of both radiative and non-radiatve transitions from the lower exciton state and hence substantial elongation of the singlet excited state lifetime^47–49^. Alternatively, the observed ABDA reaction could be due to the formation of superoxide and other downstream ROS rather than singlet oxygen. To this end, fluorescence of Ce6 could be quenched by electron transfer from the Ce6 singlet state to the iron on the SPION surface with formation of a charge-separated state, which was subsequently oxidized by molecular oxygen.

### Cytotoxicity and phototoxicity of Ce6-SCs

To ensure that Ce6-SCs can be safely injected into mice, the cytotoxicity of the Ce6-SCs, without irradiation, was examined in an MTS cell proliferation assay. Increasing concentration of Ce6-SCs were incubated with cells for 24 hours. It was found that the Ce6-SCs exhibited no obvious cytotoxicity to 4T1 murine breast cancer cells or human umbilical vein endothelial human embryonic (HUVEC) cells, even at relatively high concentrations of Fe (up to 400 ug/mL) and Ce6 (up to 300 ug/mL), respectively (Fig. 2C). Cellular uptake of Ce6-SCs was monitored by fluorescence microscopy, where a time-dependent increase in fluorescence intensity was observed at different time intervals from 0.5 to 24 h (Fig. S5, Supplementary information).

The phototoxicity of Ce6-SCs was also examined with 4T1 cells as a function of Ce6-SC concentration using MTS assays (Fig. 2D). Increasing the concentration of Ce6-SCs led to a direct increase in phototoxicity, upon irradiation with a 665 nm laser (5 J/cm^2^), with less than 10% viability observed at a dose of just 10 μg/mL. Ce6-SCs alone did not induce any significant toxicity to 4T1 cells, nor did irradiation alone. Ce6-SCs also exhibited a statistically significant improvement in phototoxicity compared with PpIX-SCs, when irradiated at their respective peak absorbances (Fig. S6, Supplementary information).

### Evaluation of Ce6-SCs in a murine tumor model

**Figure 3:**
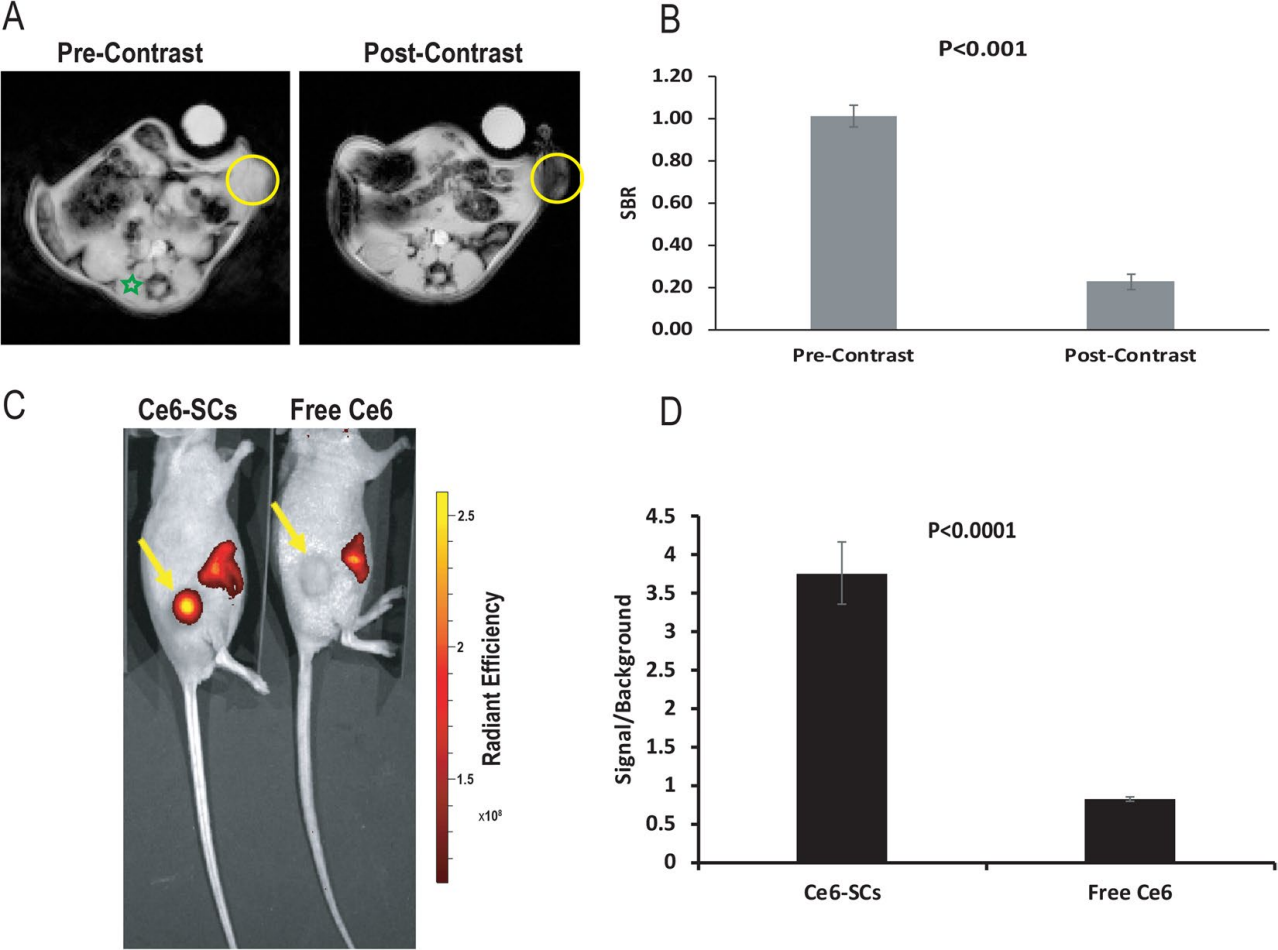
(**A**) *In vivo* MR images of mice bearing 4T1 flank tumors pre- and 24 h post-intravenous injection (i.v.) of Ce6-SCs. Images were acquired using 4.7 T MRI. Yellow circles denote the location of the flank tumor. Localization of Ce6-SCs in the tumor results in decreased intensity in the post-injection MR images. (**B**) Signal-to-background ratio (SBR) measurements were made using the candidate tumor and the paraspinous musculature (green star) as background. Quantification of the pre-injection versus post-injection SBR measurements is shown. (**C**) Representative fluorescent images of mice injected with Ce6-SCs (left) and free Ce6 (right), dosed at 2.5 mg/kg based on Ce6 weight. Fluorescent images were acquired 24 hours following i.v. injection. (**D**) Signal-to-background ratio of mice injected with Ce6-SCs and free Ce6 at 640 nm excitation and 720 nm emission. The unpaired *t*‐test was used for analyses. Statistical significance was defined as *P* < 0.05.

To demonstrate the contrast-enhancing capabilities of Ce6-SCs in a murine tumor model, 4T1 breast cancer cells were implanted subcutaneously in the flank of athymic nude mice. *In vivo*, MR imaging was then conducted before and 24 h after intravenous injection of Ce6-SCs to track their accumulation within tumors. As shown in Fig. 3A, a significant loss in signal (i.e., hypointensity) was observed in the flank tumors following injection, consistent with the accumulation of SPIONs. Signal-to-background (SBR) measurements were performed using the tumor and the paraspinous musculature as background. The post injection SBR was significantly lower at 0.23 ± 0.04 compared to the pre-injection SBR of 1.01 ± 0.05, p < 0.001 (Fig. 3B).

The Ce6-SCs within the tumor were also detectable via fluorescence imaging (Fig. 3C), despite the significant degree of quenching in the stock formulation. The signal was significantly higher than observed following the intravenous injection of free Ce6, confirming that the accumulation of Ce6-SCs in tumors is much more efficient than that of free Ce6.

**Figure 4:**
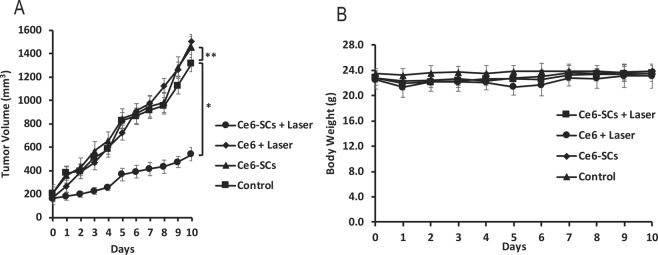
(**A**) Average tumor volume is plotted relative to the number of days after treatment. Tumor volume was measured daily. The error bars represent the standard deviations of 5 mice per group. *p < 0.001 and **p < 0.76 (unpaired *t* test). (**B**) The body weight of 4T1 tumor-bearing mice was monitored after treatment.

Next, we investigated the efficacy of PDT on the inhibition of tumor-cell growth in athymic mice bearing 4T1 flank tumors. Tumors were allowed to grow to sizes of 5–6 mm in diameter prior to PDT treatment. Mice (5–7 mice/group) were divided into four treatment groups (i) Ce6-SCs + PDT; (ii) free Ce6 + PDT; (iii) Ce6-SCs alone; and (iv) untreated controls. Ce6-SCs and free were intravenously injected at the same Ce6 concentration (2.5 mg/kg) then irradiated with a 665 nm laser at a power density of 75 mW/cm^2^ to a dose of 135 J/cm^2^ (30 min), 24 h post-injection. Tumor volume was measured daily post-irradiation for a period of 10 days. The rate of tumor growth was significantly slowed in mice that received Ce6-SCs + PDT compared with mice that received free Ce6 + PDT (day 10, P < 0.001) (Fig. 4A). There was no a significant loss of weight in any of the treatment groups following PDT (Fig. 4B). As expected, the Ce6-SCs were nontoxic in their native state but damaged target tumor cells when irradiated.

In conclusion, we successfully developed a novel nanoformulation by coating nanoclusters of SPIONs with the photosensitizer Ce6. The method is simple, efficient, only requires the use of two clinically-used compounds and is highly reproducible. The synthesized nanoclusters were systemically characterized *in vitro* and *in vivo*. The nanoclusters possess high solubility and stability in serum and good biocompatibility, thus facilitating its use biomedical applications, particularly for cancer theranostics. Ce6-SCs showed high Ce6 and SPION encapsulation efficiency and high Ce6 payloads. The Ce6-SCs also exhibited enhanced delivery of Ce6 into tumors, compared with free Ce6, and could be visualized by both MR and fluorescence imaging. The therapeutic activity of Ce6 molecules in Ce6-SCs was minimally reduced when incorporated onto SPION nanoclusters and exhibited efficient phototoxicity in tumor-bearing mice because of the enhanced delivery into the tumor, compared with free Ce6. We believe that based on our findings Ce6-SCs represent a promising theranostic agent for clinical translation.

## Methods

### Materials

Chlorin e6 (Ce6, MW = 596.68) was purchased from Santa Cruz Biotechnology, iron (III) acetylacetonate, 1,2-hexadecanediol, benzyl ether, oleylamine, and anhydrous dimethyl sulfoxide (DMSO) were obtained from Aldrich. Oleic acid was purchased from Chem-Impex International, Inc. Ethanol was purchased from Decon Labs, Inc. All other chemicals and solvents were of analytical grade and were used without further purification. All the buffer solutions were prepared with deionized water.

### Synthesis of SPIONs

SPIONs (Fe_3_O_4_) were synthesized according to thermal decomposition method as previously described^43^. Briefly, iron(III) acetylacetonate [Fe(acac)_3_] (2 mmol), 1, 2-hexadecanediol (5 mmol), oleic acid (2 mmol), and oleylamine (6 mmol) were added, to benzyl ether (20 mL) in a two-necked round-bottomed flask, equipped with a reflux condenser and stir bar. The mixture was heated to 200 °C for 15 min, and then the temperature of the mixture was maintained at 300 °C for 1 h under nitrogen with vigorous stirring. At the end of this period, the mixture was left to cool down to room temperature, two volumes of ethanol were added, and the resulting mixture was centrifuged (5500 × g for 15 min) to precipitate the nanoparticles. The particles were then allowed to air dry and dissolved in toluene. Large aggregates were removed by centrifugation at 3000 × g for 15 min.

### Preparation of Ce6-SCs

A mixture (200 μL) containing Ce6 (2 mg into dimethyl sulfoxide (DMSO)) and SPION (1, 2, 4, 5, or 6 mg based on the Fe concentration in toluene) was pipetted into a glass vial containing 4 mL of water, and the sample was sonicated until a homogeneous solution was observed. The toluene was evaporated overnight. Dialysis was performed with dialysis tubing (3500 MWCO, Fisherbrand) into 4L of water to remove dimethyl sulfoxide. The Ce6-SCs were further purified by MACS (25 LD columns, Miltenyi Biotec, Germany) column.

### Characterization of Ce6-SCs

The diameter and size distributions of the Ce6-SCs were measured with dynamic light scattering (DLS, Malvern, Zetasizer, Nano-ZS). The morphology of the nanoparticles was observed using a transmission electron microscope (TEM) (JOEL 1010). T_2_ relaxation times were measured using a benchtop relaxometer (Bruker, mq60 NMR analyzer). The encapsulation efficiency and payload of Ce6 was determined using UV-Vis spectrophotometer (Varian, 100 Bio). The concentration of the coated Ce6 was quantified by dissolving the Ce6-SCs in DMSO, measuring the absorbance at 404 nm and comparing the reading to a standard concentration curve of free Ce6 in DMSO. Iron concentration was quantified by plasma optical emission spectroscopy (ICP-OES) (Spectro Genesis, GMBH).

### Ce6-SCs release and stability studies

The amount of Ce6 adsorbed on Ce6-SCs, size stability and magnetic properties (T_2_ mode) in water/fetal bovine serum (FBS) (1/10, v/v) solution were determined as previously reported^43^. Briefly, prepared Ce6-SCs were incubated in water as well as in FBS at 37 °C for the stability study. DLS was used to monitor the particle size for 6 days. Aliquots taken from the sample were tested at various time points for the determination magnetic properties via T_2_.

To investigate *in vitro* release behavior of Ce6 from the nanocluster, 1 ml Ce6-SCs solution was placed in a tube containing 9 ml FBS and incubated at 37 °C under continuous shaking. At specific time intervals within two days, 0.5 ml solution was sampled and run through MACS columns (25 LD columns, Miltenyi Biotec, Germany) to separate free Ce6 released from Ce6 nanoclusters. UV-absorption spectroscopy was used to determine the amount of Ce6 in the purified Ce6 nanocluster samples. Normalized peak absorbance was measured over 2 days and the cumulative release percentage of Ce6 was plotted as a function of incubation time.

### MTS Assay

Human umbilical vein endothelial (HUVEC) and 4T1 cells (1 × 10^4^ cells per well) were seeded in 96-well plates and incubated overnight to allow the cells to attach to the surface of the wells. The cells were then mixed with increasing concentrations of Ce6-SCs for 24 h, and the cell viabilities were determined using an MTS assay (Abcam Inc.) according to the supplier’s instructions. Briefly, after 24 h of incubation with Ce6-SCs, 10 ul of MTS reagent was added. After 2 h, absorbance was read at 490-nm on a Tecan microplate reader.

### Phantom and animal imaging by MR

All methods involving animals in this manuscript were carried out in accordance with relevant guidelines and regulations. All experimental protocols were approved by the Institutional Animal Care and Use Committee of the University of Pennsylvania. Relaxometry measurements were performed in T_2_* mode (Varian, 4.7T); Iron concentration was determined by ICP-OES. A plastic 384-well plate (MR phantom) was used to test the T_2_ hypointensity associated with Ce6-SCs compared to control (i.e., water) on a 4.7T magnet. [Fe] concentrations were as follows: 0.5, 0.25, 0.125, 0.0625, and 0.0312 × 10^−3^ m. Unenhanced MR images of the mice bearing 4T1 flank xenografts were first obtained, and then MRI was performed 24 h after the intravenous injection of Ce6-SCs at a dose of 2.5 mg kg^−1^ (based on Fe mass) via retro-orbital injection. The MR acquisition parameters were as follows: TR 200 ms, TE 5 ms, matrix 128 × 128, and gap 0. Contrast enhancement on T_2_*-weighted imaging (seen as hypoenhancement following injection of Ce6-SCs) was quantified using ImageJ. The region of interest of tumor (signal) was normalized to the paraspinous musculature as background and expressed as a signal-to-background ratio (SBR). T-test comparisons were made between the mean SBRs in animals pre-injection versus post-injection, with a p-value of <0.05 considered to be statistically significant.

### Phantom and animal imaging by Fluorescence

Serial 1/2× dilutions from [40 μg mL^−1^] to 1/32 × [1.25 μg mL^−1^] of Ce6 in DMSO/water (1/20, v/v) solution were used in a 96-well plate as a control. 5% DMSO was necessary to improve the solubility of Ce6 in water. An equivalent amount of Ce6-SCs was dissolved in the same solution for comparison. For the *in vivo* animal experiments, 4T1 tumor cells were introduced into female nude mice. When the tumor volume reached about 80 mm^3^, Ce6-SCs nanoparticles (dose: equivalent Ce6 2.5 mg/kg) and free Ce6 (dose: 2.5 mg/kg) were injected intravenously into the tumor-bearing nude mice. Images were acquired with a Perkin Elmer IVIS Spectrum *In Vivo* System (excitation, 640 nm; emission, 720 nm; exposure time, Auto; binning 4; and *f* = 2).

### *In vitro* PDT

For *in vitro* PDT experiments, 4T1 cells (1 × 10^4^ cells) were seeded onto 96-well cell culture plates and incubated for 24 h with various concentrations of Ce6-SCs (0.625, 1.25, 2.5, 5, and 10 ug/mL based on the Ce6 concentration). The cells were then irradiated with a 665 nm diode laser (B&W Tek, Inc.) to a dose of 5 J/cm^2^ (16.40 min), while the control groups were still cultured in the dark. Illumination was delivered through microlens-tipped fibers (Pioneer), and intensity of laser output was monitored and adjusted (LabMaster power meter; Coherent) to a power density of 5 mW/cm^2^. Afterward, all samples were incubated in the dark for another 24 h. Cell viability was measured by the MTS colorimetric assay and recorded as the percentage of live cells in the treated samples compared with the percentage of live cells in the untreated control.

### *In vivo* PDT of Ce6-SCs in 4T1 tumor-bearing mice

4T1 tumors cells (5 × 10^6^ cells in 100 ul) were implanted in the right flank of athymic nude female mice (aged 6 weeks). When the tumor size reached approximately 80 mm^3^, free Ce6 (2.5 mg/kg of Ce6), and Ce6-SCs (2.5 mg/kg of Ce6) were injected into the mice via retro-orbital injection. At 24 h post-injection, mice were irradiated (30 min) at the tumor site with 665 nm light (B&W Tek, Inc. diode laser) to a dose of 135 J/cm^2^ at a power density of 75 mW/cm^2^. The therapeutic efficacy of the treatments was monitored by measuring the tumor volumes calculated as 1/2 × (length × width^2^). All measurements were made with a caliper.

## Supplementary information

Supplementary Info
