# Supplementary material for: Chlorin e6-Coated Superparamagnetic Iron Oxide Nanoparticle (SPION) Nanoclusters as a Theranostic Agent for Dual-Mode Imaging and Photodynamic Therapy
Source: Sci Rep. 2019 Feb 22;9:2613. doi: 10.1038/s41598-019-39036-1 (PMC6385362; doi:10.1038/s41598-019-39036-1)
Supplement: Supplementary file 1 — Supplementary Info [file 41598_2019_39036_MOESM1_ESM.pdf]

Supplementary information

Chlorin e6-Coated Superparamagnetic Iron Oxide Nanoparticle (SPION) Nanoclusters as a Theranostic Agent for Dual-Mode Imaging and Photodynamic Therapy

Ahmad Amirshaghaghi[1], Lesan Yan[1], Joann Miller[2], Yonathan Daniel[3], Joel M. Stein[1,4], Theresa M. Busch[2], Zhiliang Cheng[1], Andrew Tsourkas[1,*]

[1]Dr. A. Amirshaghaghi, Dr. L. Yan, Prof. Z. Cheng, Prof. A. Tsourkas
Department of Bioengineering
University of Pennsylvania
Philadelphia, PA 19104, USA
[*]E-mail: atsourk@seas.upenn.edu
[2]J. Miller, Prof. T. M. Busch
Department of Radiation Oncology
Perelman School of Medicine
University of Pennsylvania
Philadelphia, PA 19104, USA
[3]Y. Daniel
Department of Biology, College of Computer, Mathematical, & Natural Sciences
University of Maryland, College Park,
Maryland 20742, USA
[4]Prof. J. M. Stein
Department of Radiology
University of Pennsylvania
PA 19104, USA

SUPPLEMENTAL TABLES:

Table S1. Repeatability of Ce6-SCs synthesis

	Batch 1	Batch 2	Batch 3	Average	St. Dev.
**Hydrodynamic Diameter (nm)**	97.03	100.7	91.42	96.38	4.67
**PDI**	0.201	0.190	0.184	0.19	0.0086
**Relaxivity (r_2) ($mM^{-1}s^{-1}$)**	215	220	247	227	17.2

Table S2. Physical-chemical properties of Ce6-SCs

Starting Ce6:Fe Ratio (w/w)	2:1	1:1	1:2	1:2.5	1:3
**Encapsulation Efficiency (%)**	65.39	71.06	81.07	90.87	100
**Ce6 Payload: Ce6/(Ce6+Fe) (%)**	56.52	41.52	28.35	26.11	24.39
**Ce6 Payload: Ce6/(Ce6+Fe+O) (%)**	52.43	34.04	18.35	14.36	11.21
**Relaxivity (r_2) ($mM^{-1}s^{-1}$)**	247	301	369	382	410
**Hydrodynamic Diameter (nm)**	91.42	90.03	92.6	96.8	114.4
**PDI**	0.184	0.165	0.147	0.158	0.156

SUPPLEMENTAL FIGURES:

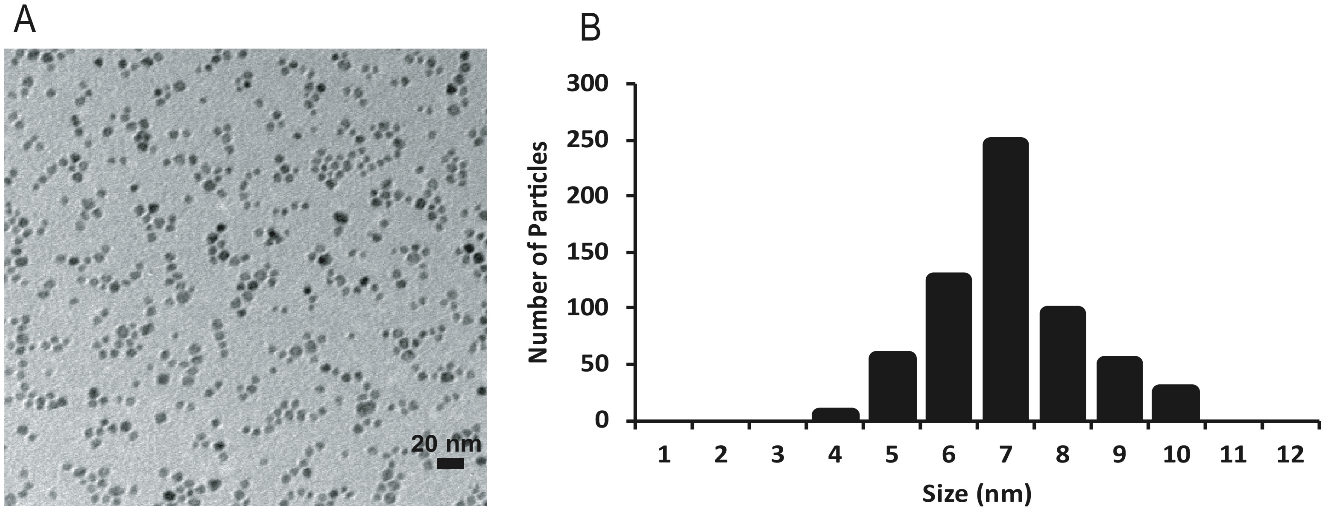

Figure S1. (A) Transmission electron microscopy (TEM) of superparamagnetic iron oxide nanoparticles (SPIONs) following thermal decomposition reaction. Scale bar = 20 nm. (B) Analysis of TEM results demonstrating the size distribution of SPIO (diameter = 7.6 ± 1.0 nm).

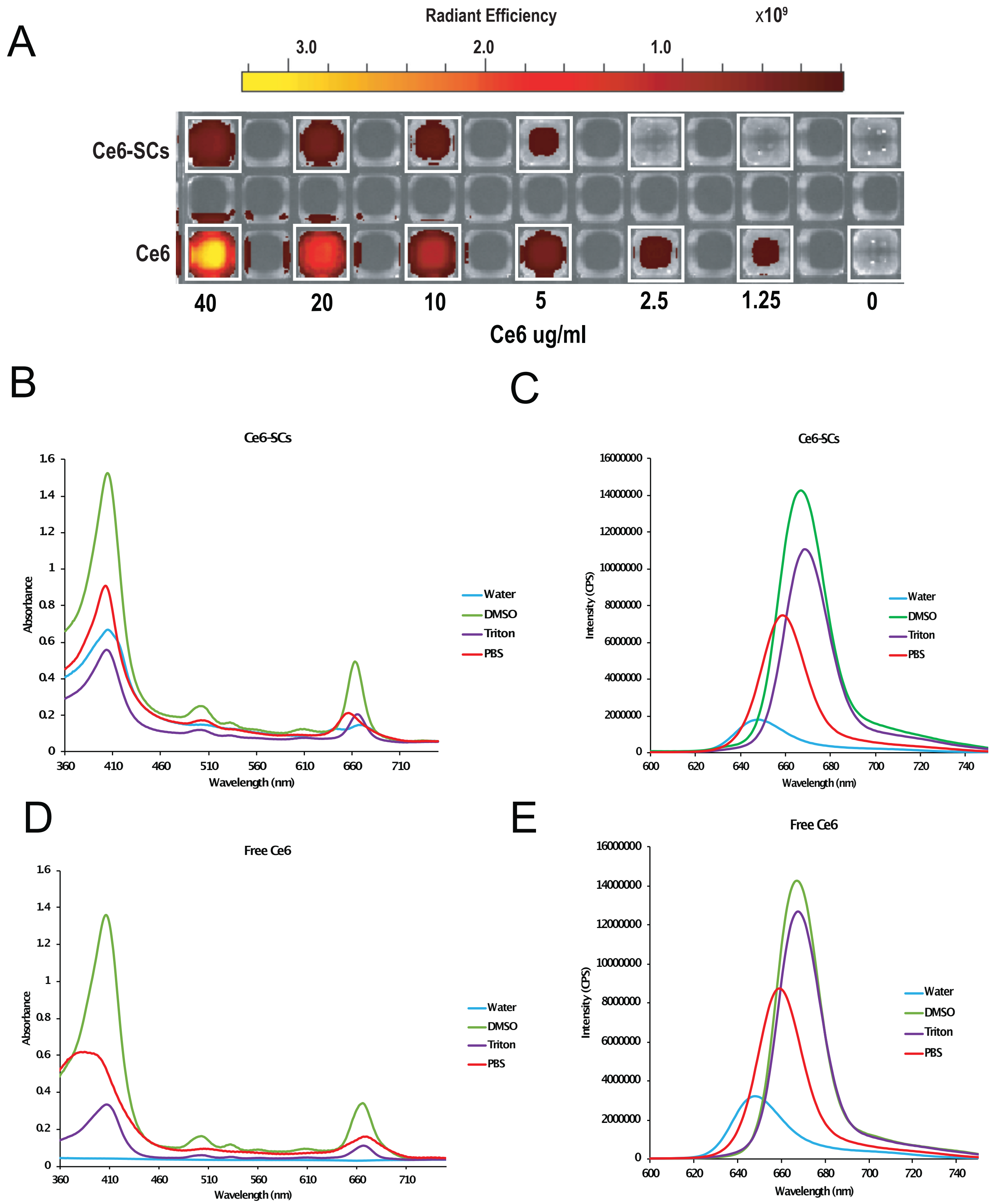

Figure S2. (A) Fluorescent image of a microplate containing increasing concentrations of Ce6-SCs (top row) and Ce6 free (bottom row) in 5% DMSO/95% water. Instrumentation/parameters: Perkin Elmer IVIS Spectrum In Vivo System (excitation 640 nm, emission 720 nm, exposure time= Auto, binning 4, f=2). UV-vis and fluorescence spectra of Ce6-SCs (B), (C) and free Ce6 (D), (E) in water, DMSO, 1% Triton X-100, and PBS with equal Ce6 concentration (5 μM).

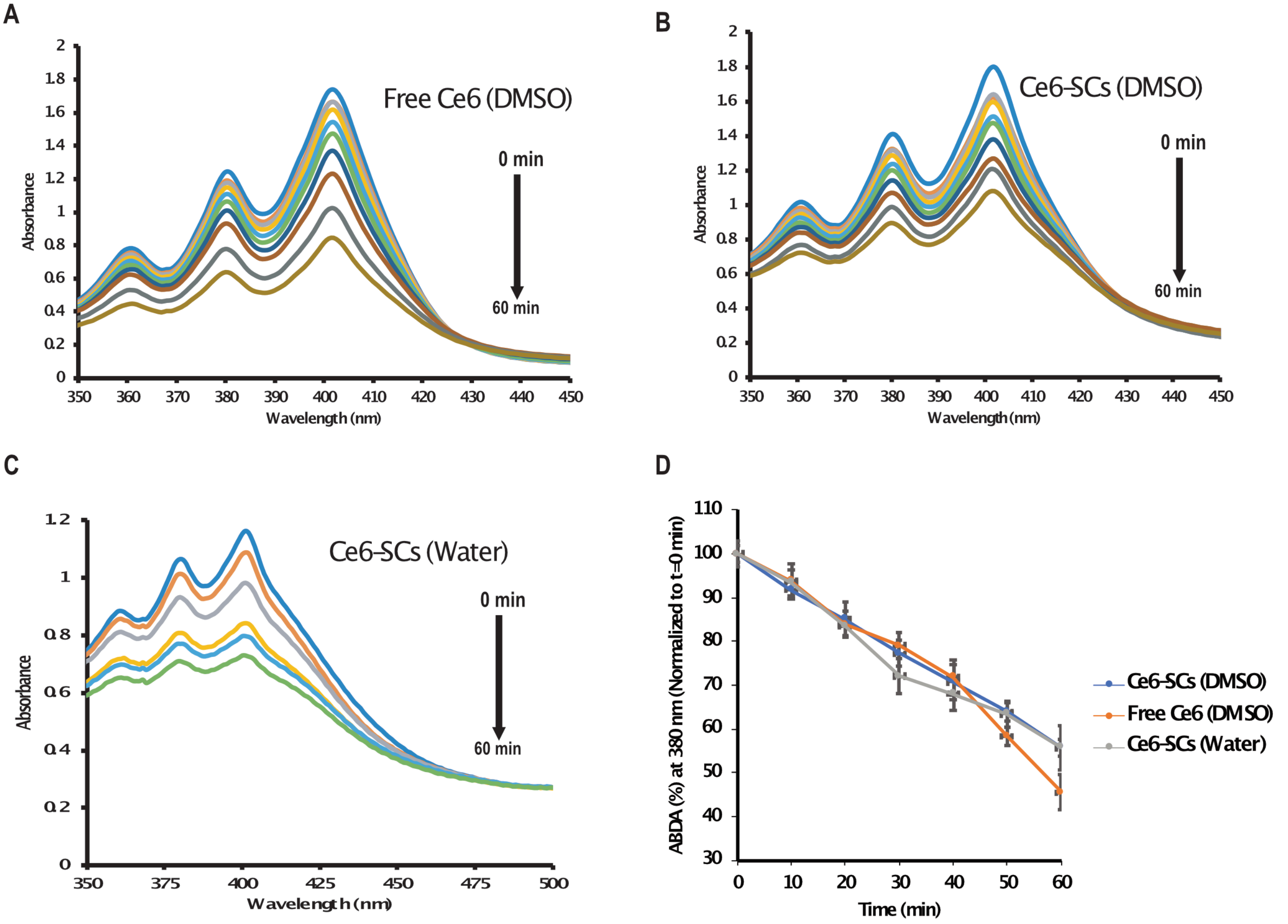

Figure S3. Comparison of the singlet oxygen generation rate of various Ce6 formulations. The absorption spectra of 9,10-Anthracenediyl-bis-(methylene)dimalonic acid (ABDA, 30 uM) after photodecomposition by ROS generation in the presence of A) Free Ce6, B) Ce6-SCs in DMSO and C) Ce6-SCs in water at the same dose of Ce6 (10uM) for different exposures from 0 to 60 min. The method for $^1O_2$ detection by UV-vis spectroscopy was based on the protocol reported previously.[3] Briefly, different Ce6 formulations (Ce6= 10 uM) were prepared in 15 mL DMSO and water in presence of 30 uM 9,10-Anthracenediyl-bis-(methylene)dimalonic acid (ABDA). Then the solutions were irradiated under a laser (λ =665 nm, power density = 5 mW/cm^2), and aliquots of sample solution were removed from the irradiated sample at predetermined intervals and subjected to UV−vis absorption measurement. The absorbance change of ABDA at 350-500 nm under different laser-irradiation periods was monitored. (D) Percentage of ABDA at 380 nm versus light irradiation time.

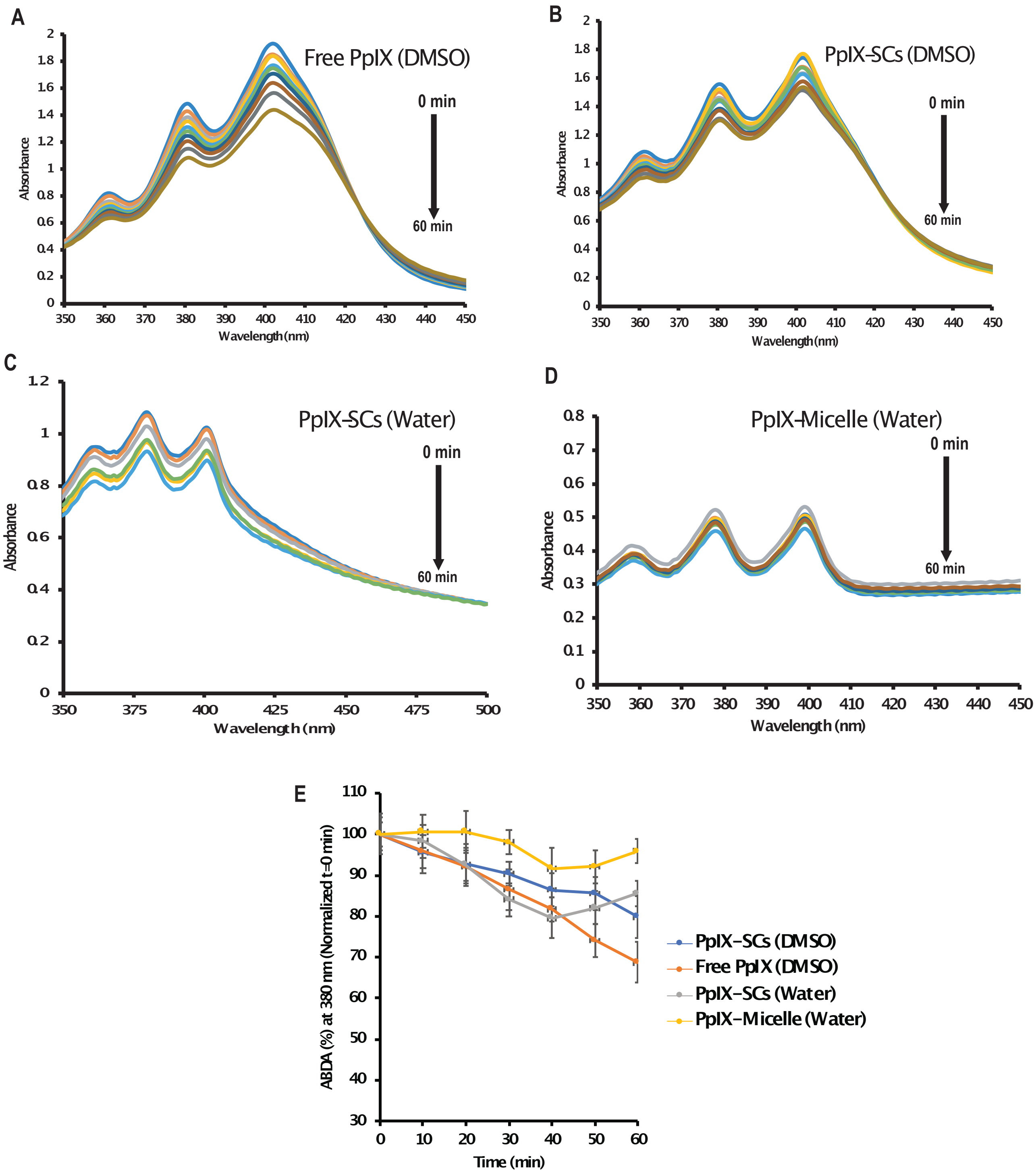

Figure S4. The same experiment (as mentioned in Figure S3) was applied to free PpIX (A), PpIX-SCs (B) in DMSO and PpIX-SCs (C), PpIX micelle (D) in water. The solutions were irradiated under a laser ($\lambda = 632$ nm, power density = 5 mW/cm^2). (E) Percentage of ABDA at 380 nm versus light irradiation time.

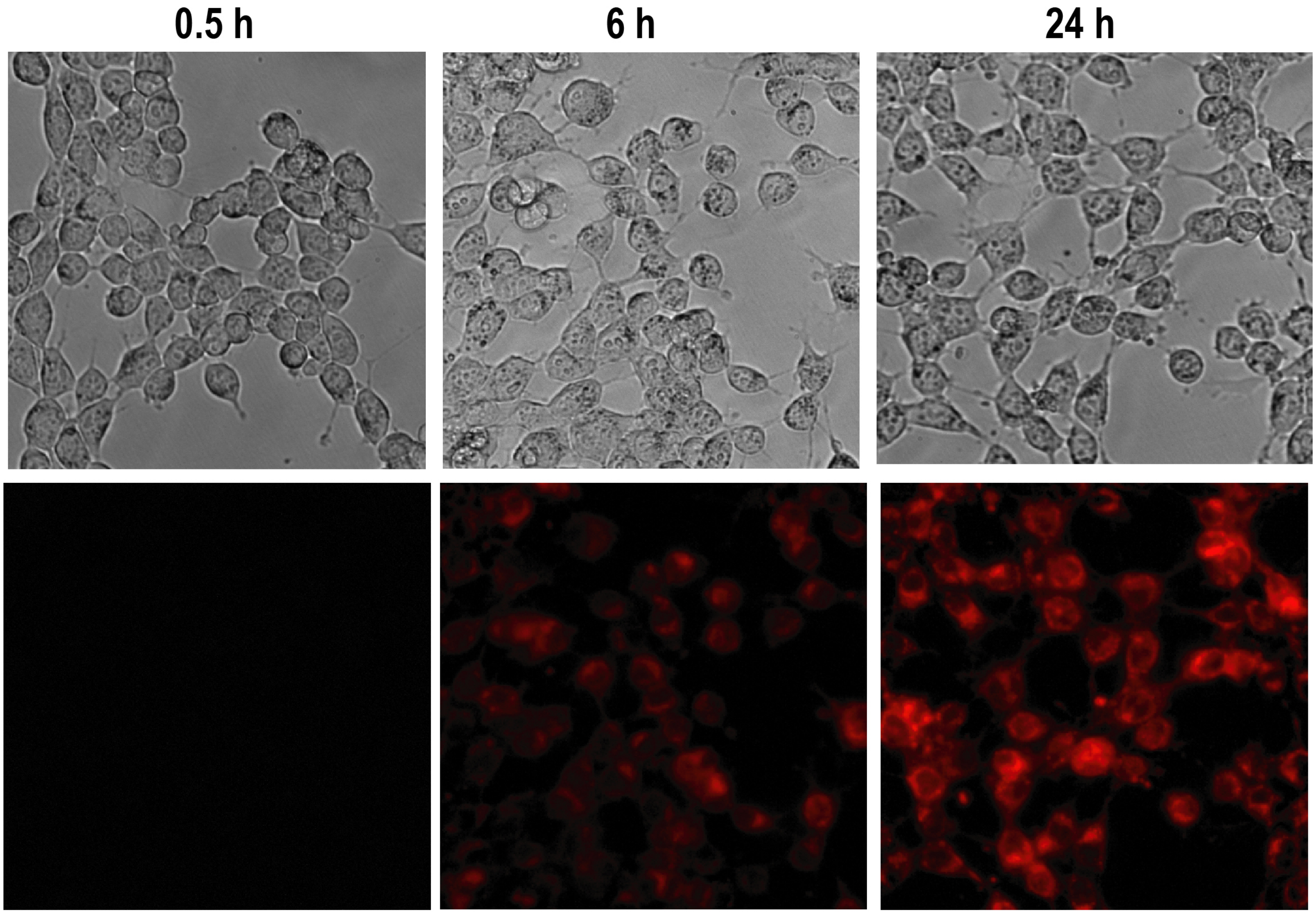

Figure S5. Phase contrast (top row) and fluorescence microscopy (bottom row) images of 4T1 cells incubated with Ce6-SCs for 0.5, 6, and 24 h.

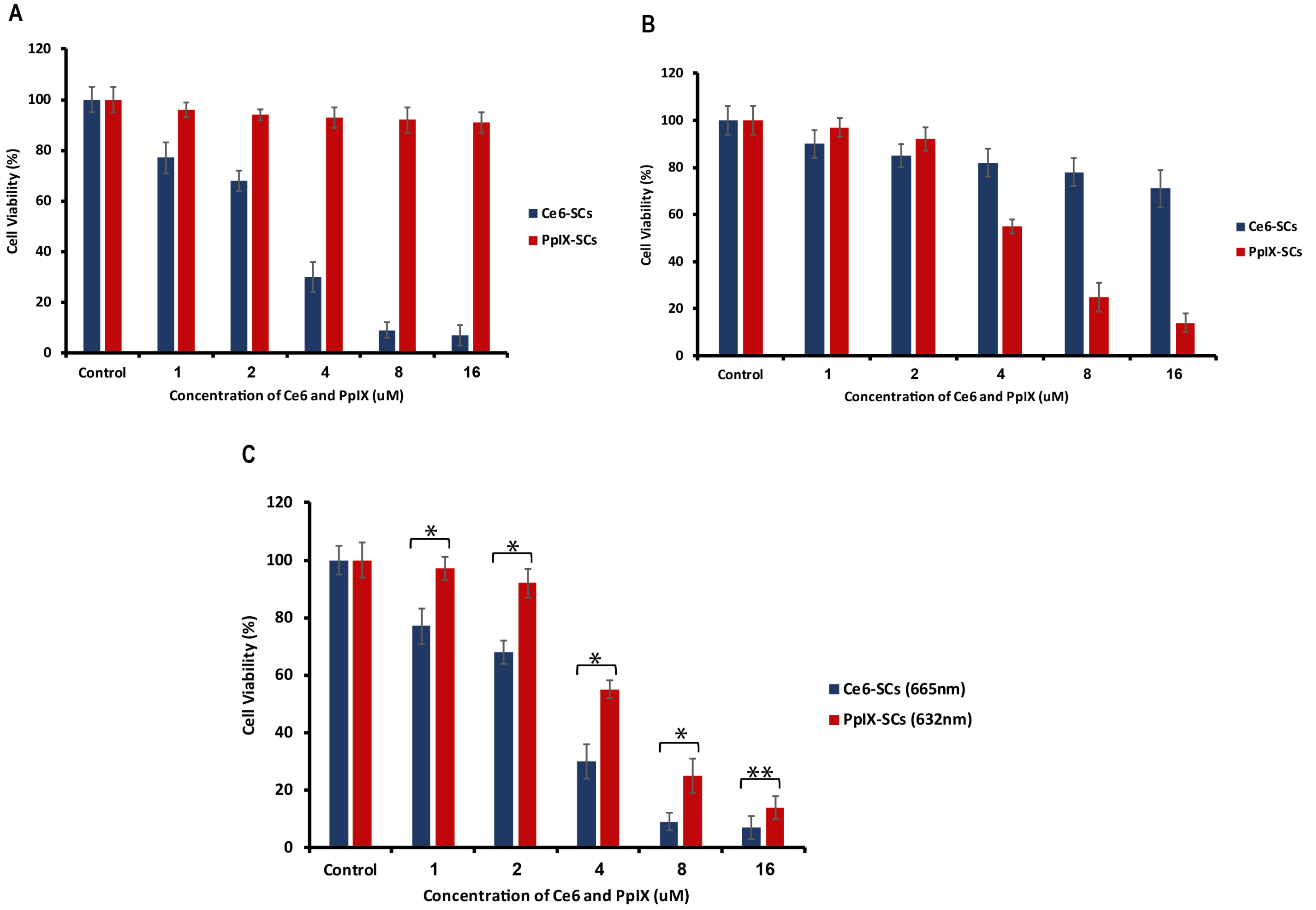

Figure S6. Viability of 4T1 cells treated with different concentrations of Ce6-SCs and PpIX-SCs and irradiated with a (A) 665 nm and (B) 632 nm laser. These wavelengths correspond to the absorbance peak of each PS. (C) Comparison of 4T1 cell viability when treated with Ce6-SCs + 665 nm irradiation or PpIX-SCs + 632 nm irradiation (* $P<0.0001$ and ** $P<0.001$). All studies were performed using a power density = 5 mW/cm^2.
